# Identification and Functional Analysis of a Pseudo-Cysteine Protease from the Midgut Transcriptome of *Sphenophorus levis*

**DOI:** 10.3390/ijms222111476

**Published:** 2021-10-25

**Authors:** Priscila Yumi Tanaka Shibao, Milene Ferro, Fernando Fonseca Pereira de Paula, Bruno Salata Lima, Flávio Henrique-Silva

**Affiliations:** Laboratory of Molecular Biology, Department of Genetics and Evolution, Federal University of São Carlos, Rodovia Washington Luis, Km 235, São Carlos 13565-905, SP, Brazil; priscila.shibao@gmail.com (P.Y.T.S.); milenef@gmail.com (M.F.); fonseca.fpp@gmail.com (F.F.P.d.P.); brunos_l@hotmail.com (B.S.L.)

**Keywords:** pseudoenzyme, cysteine protease, coleoptera, protease inhibitor, cysteine protease, insect digestion

## Abstract

The *Sphenophorus levis* (Coleoptera, Curculionidae) is one of the main pests of sugarcane in Brazil. Although its major digestive proteases are known, its complex digestive process still needs to be further understood. We constructed a transcriptome from the midgut of 30-day-old larvae and identified sequences similar to its major digestive protease (cysteine cathepsin Sl-CathL), however, they presented a different amino acid than cysteine in the active cleft. We identified, recombinantly produced, and characterized Sl-CathL-CS, a pseudo cysteine protease, and verified that higher gene expression levels of Sl-CathL-CS occur in the midgut of 30-day old larvae. We reverted the serine residue to cysteine and compared the activity of the mutant (Sl-CathL-mutSC) with Sl-CathL-CS. Sl-CathL-CS presented no protease activity, but Sl-CathL-mutSC hydrolyzed Z-Phe-Arg-AMC (V_max_ = 1017.60 ± 135.55, K_m_ = 10.77 mM) and was inhibited by a cysteine protease inhibitor E-64 (K_i_ = 38.52 ± 1.20 μM), but not by the serine protease inhibitor PMSF. Additionally, Sl-CathL-CS interacted with a sugarcane cystatin, while Sl-CathL-mutSC presented weaker interaction. Finally, protein ligand docking reinforced the differences in the catalytic sites of native and mutant proteins. These results indicate that Sl-CathL-CS is a pseudo-cysteine protease that assists protein digestion possibly by interacting with canecystatins, allowing the true proteases to work.

## 1. Introduction

The sugarcane weevil *Sphenophorus levis* (Coleoptera; Curculionidae) is one of the main pests of sugarcane crops (*Saccharum* spp.) in Brazil. *S. levis* is a particularly challenging pest to control because the larvae develop inside the stem, opening galleries inside the plant, making the use of commercial pesticides inefficient [1,2]. Therefore, there is a need for alternative control strategies, which requires a deep understanding of the insect’s digestive tract.

Coleoptera insects mainly use cysteine proteases as digestive enzymes [3,4,5,6,7], while Lepidoptera usually rely on serine proteases activities [8,9]. Accordingly, the digestive protease profile of *S. levis* has revealed that its major protease is Sl-CathL, a cysteine cathepsin L-like, followed by other proteases, e.g., trypsin-like enzymes [5]. Additionally, other enzymes show complementary activity in the insect’s digestion. Maltase and amylase are responsible for starch digestion [2,5], invertases are important for the hydrolysis of disaccharide sucrose into monomers of fructose and glucose [10], and pectinases for the degradation of the plant cell wall during insect feeding [11,12]. Meanwhile, sugarcane produces cystatins, which are tight-binding cysteine protease inhibitors that show high inhibitory capability against Sl-CathL [13]. This relationship between insect proteases and plant protease inhibitors is well established and is part of a co-evolution process that also involves local fauna and is mainly regulated by the panel of inhibitors in a specific niche [14].

When analyzing the transcriptome from the midgut of *S. levis* larvae, one sequence was found to be related to Sl-CathL, although it presented a serine residue instead of cysteine in the putative protease active site. A deeper analysis revealed some other sequences that presented amino acids other than serine in the putative catalytic site. Some proteins, called pseudoenzymes, share homology with other proteases but present at least one amino acid in the catalytic site different from the conserved protease, resulting in a protein without the original enzymatic activity [15]. Early reports described their role as regulators of intracellular processes, cellular signalization, and allosteric enzyme activity [15,16,17], but lately, it has been demonstrated that pseudoenzymes may be involved in the physiological function of insects [18]. 

In this regard, we decided to investigate the role of the pseudo-cysteine protease Sl-CathL-CS in the insect digestive process. We identified the expression pattern in developmental stages of *S. levis* and the insect tissues, then cloned, expressed, and characterized the pseudo-cysteine protease (Sl-CathL-CS) and the mutant (Sl-CathL-mutSC), in which we reverted the Ser^138^ to Cys^138^ and analyzed their interaction with CaneCPI-1 [19], a sugarcane cystatin.

## 2. Results

### 2.1. Sphenophorus levis Midgut Transcriptome and Identification of Putative Pseudo-Cysteine Proteases

The transcriptome information of pooled RNA from 30-day-old *S. levis* larvae is summarized in Table 1. The de novo assembly quality control was performed using BUSCO [20], which revealed that 96% of genes were complete (single copy and duplicate), 1% of genes (12) were fragmented and 3% of the assembled genes (44) were missing.

### 2.2. Identification of Putative Proteases

Initially, we obtained approximately 1220 sequences similar to Sl-CathL. Then, we discarded sequences that shared less than 40% identity with Sl-CathL, to exclude fragments and sequences that were too short and did not present His^272^ from the putative catalytic site. The final sequences were divided in three groups, according to the shared identity with Sl-CathL, as follows: group 1, 99 to 75% shared identity; group 2, between 74 and 50% and group 3, between 49 and 30% (Supplementary Figure S1).

**Figure 1:**
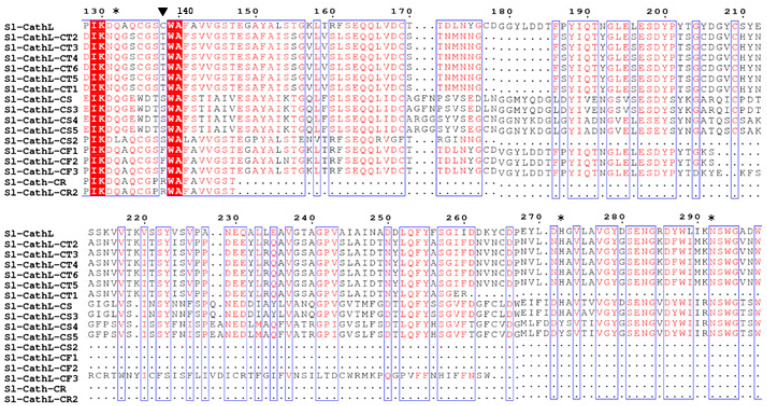
Variants of Sl-CathL presenting residue other than cysteine in position 138. Sequences were cropped to show mutation in the putative catalytic site, and were numbered starting with the initiating methionine of Sl-CathL. Black triangle indicates position 138 and asterisks (*) indicate residues forming catalytic triad and Gly^132^, which is pivotal for correct conformation of substrates during catalysis. Residues colored in red have high consensus, and those in blue are the most variable. Identical residues are highlighted in red, and blue box indicates similar parts of protein sequence.

In groups 2 and 3, there were sequences in which Cys^138^ was replaced by another amino acid residue, such as cysteine, serine, threonine, or phenylalanine (Figure 1). Among these variants, Sl-CathL-CS had 47% shared identity and was included in group 3. For this analysis, we considered all sequences retrieved in the transcriptome to provide the complete repertoire of variants.

### 2.3. Relative Expression of Sl-CathL-CS in Different Parts of the Larvae

**Figure 2:**
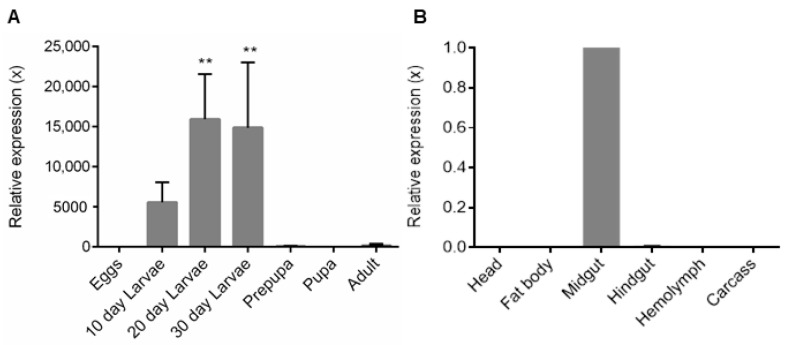
Relative expression of Sl-CathL-CS in developmental stages and parts of *S. levis*. (**A**) Sl-CathL-CS expression is higher in 20- and 30-day-old larvae. We used pupa expression levels as control, as it shows the lowest expression. (**B**) Relative expression of Sl-CathL-CS in different parts of 30-day-old larvae, using midgut as control. GAPDH was used as internal control for both analyses. ** *p* < 0.001.

The Sl-CathL-CS expression was assessed in the developmental stages of the insect (eggs; 10-, 20- and 30-day-old larvae; prepupa; pupa; and adult) and in different parts of the 30-day-old larvae (head, fat body, hindgut, midgut, hemolymph, and integument). Among the developmental stages, Sl-CathL-CS showed high expression in 30-day-old larvae, and this stage was used for further analysis regarding the gene expression levels in the tissues. This stage shows higher protease and hydrolase activity. The pseudoenzyme was expressed mainly in 20- and 30-day-old larvae and the midgut and hindgut, but with very low expression in the latter (Figure 2). Sl-CathL-CS expression levels in the head, hemolymph, and carcass were null and the level in the fat body was practically irrelevant. An RT-qPCR analysis compared the gene expression levels with GADPH in the referenced developmental stages and tissues. Agarose gel (1.5%) of the RNA used for RT-qPCR is available in Supplementary Figure S2, and the melting curves are presented in Supplementary Figure S3. 

### 2.4. Heterologous Expression of Sl-CathL-CS and Sl-CathL-mutSC

**Figure 3:**
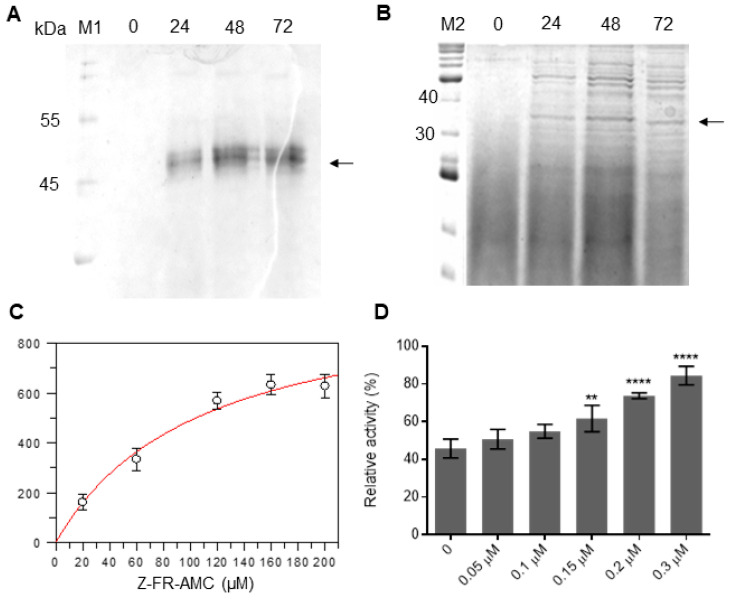
Sl-CathL-CS and Sl-CathL-mutSC expression and characterization. Recombinant expression of (**A**) Sl-CathL-CS and (**B**) Sl-CathL-mutSC according to time. M1: Wide-range molecular weight marker (Sigma-Aldrich, St. Louis, MO, USA), and M2, BenchMark protein (Thermo Fisher, Waltham, MA, USA). Black arrows on right indicate the proteins. (**C**) Sl-CathL-mutSC activity on cysteine protease substrate Z-Phe-Arg-AMC. Assay carried out in triplicate and standard deviations are indicated with bars. (**D**) Competition assay with addition of Sl-CathL-CS. Sl-CathL was used as protease and Z-Phe-Arg-AMC as fluorogenic substrate. 0: reaction with addition of CaneCPI-1 and absence of Sl-CathL-CS. Bars represent relative activity of Sl-CathL (%) inhibited by CaneCPI-1 with the addition of Sl-CathL-CS. Numbers under graph represent quantity of Sl-CathL-CS added. Assays were conducted in triplicate and the standard deviation is indicated by bars. ** *p* < 0.001, **** *p* < 0.0001 (one-way ANOVA, followed by Dunnett’s).

The Sl-CathL-CS and Sl-CathL-mutSC presented the highest yield at 48 and 72 h of induction respectively, with 0.75% (*v*/*v*) methanol in *P. pastoris* yielding 7 and 2 mg/L. As both proteins were cloned in frame with a C-terminus 6x His tag sequence (N-AAASFLEQKLISEEDLNSAVDHHHHHH-C), they could be purified in a single step through nickel immobilized metal affinity chromatography (IMAC). An analysis in 15% SDS-PAGE revealed bands of around 45 kDa for Sl-CathL-CS (Figure 3A) and 35 kDa for Sl-CathL-mutSC (Figure 3B). 

### 2.5. Enzymatic Characterization of Sl-CathL-CS and Sl-CathL-mutSC

#### 2.5.1. Sl-CathL-CS and Sl-CathL-mutSC Protease Activity 

The Sl-CathL-CS did not display any activity on complex, specific serine and cysteine protease fluorimetric substrates. The reversion of Ser^138^ to Cys^138^ in Sl-CathL-mutSC provided the protein with the capability of degrading skim milk powder and hydrolyzing Z-Phe-Arg-AMC with K_m_ = 10.77 mM and V_max_ = 1017.60 ± 135.55 (Figure 3C), but not Z-Leu-Arg-AMC and Z-Arg-Arg- AMC. Assays with skim milk powder (complex substrate) are presented in Supplementary Figure S4. E-64, a specific cysteine protease inhibitor, was able to inhibit Sl-CathL-mutSC, with K_i_ of 38.52 ± 1.20 μM, while up to 200 µM of PMSF, a serine protease inhibitor, showed no effect on Sl-CathL-mutSC activity.

#### 2.5.2. Competition Assay

Kinetic assays with Sl-CathL, Z-Phe-Arg-AMC, CaneCPI-1 and increasing amounts of Sl-CathL-CS were conducted to evaluate the interaction between Sl-CathL-CS and CaneCPI-1. Sl-CathL proteolytic activity on Z-Phe-Arg-AMC was measured and Cane-CPI-1 was added to the reaction. After determining its relative activity in the presence of the inhibitor, increasing amounts of Sl-CathL-CS were added to the reaction and the relative activity of Sl-CathL was again calculated. Sl-CathL-CS was able to compete with Sl-CathL for the inhibitor CaneCPI-1. The more Sl-CathL-CS that was added to the reaction, the higher the observed rates of substrate degradation (Figure 3D).

### 2.6. Pull-Down

**Figure 4:**
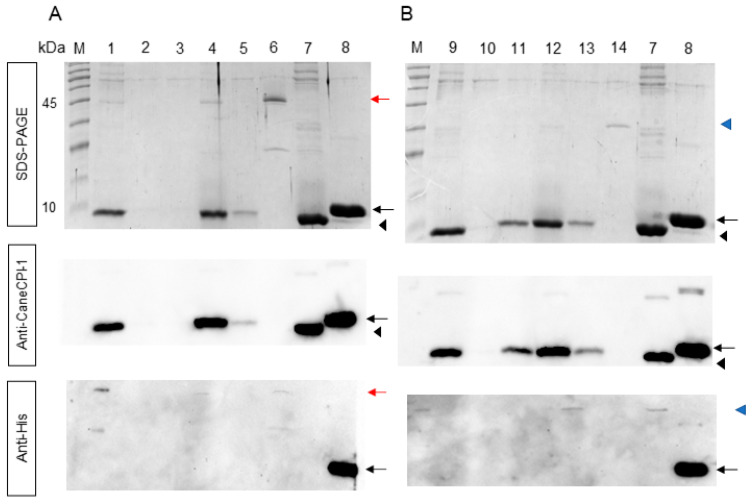
Interactions between Sl-CathL-CS, Sl-CathL-mutSC and CaneCPI-1. (**A**) Assay conducted with SL-CathL-CS. (**B**) Assay between Sl-CathL-mutSC and CaneCPI-1. M: Blue Classic Marker (Jena Biosciences, Jena, DE). 1 and 9: Flow through; 2–3 and 10–11: washing steps; 4–5 and 12–13: elution steps; 6: Sl-CathL-CS; 7: CaneCPI-1 without His-tag; 8: CaneCPI-1; 14: Sl-CathL-mutSC. Red arrow indicates Sl-CathL-CS; blue arrow indicates Sl-CathL-mutSC; black arrow indicates CaneCPI-1 with His-tag, and black triangle indicates CaneCPI-1 after His-tag removal. It is possible to see size difference before and after His-tag removal from CaneCPI-1.

The interaction between Sl-CathL-CS and Sl-CathL-mutSC with sugarcane cystatin CaneCPI-1 is shown in Figure 4. CaneCPI-1 binds with both. The interaction between CaneCPI-1 and Sl-CathL-CS (Figure 4A) is higher than that between CaneCPI-1 and Sl-CathL-mutSC (Figure 4B). Although cystatin could not be detected in the washing steps of the assay with Sl-CathL-CS (lanes 2 and 3), CaneCPI-1 was detected in lane 11. A Western blotting assay with an anti-CaneCPI-1 antibody confirmed that the band in lane 11 was CaneCPI-1, while Western blotting conducted with anti-his antibody confirmed that the elution noted in lanes 4, 5, 12, and 13 was due to interaction of CaneCPI-1 with the tested proteins.

### 2.7. Static Protein Ligand Docking

**Figure 5:**
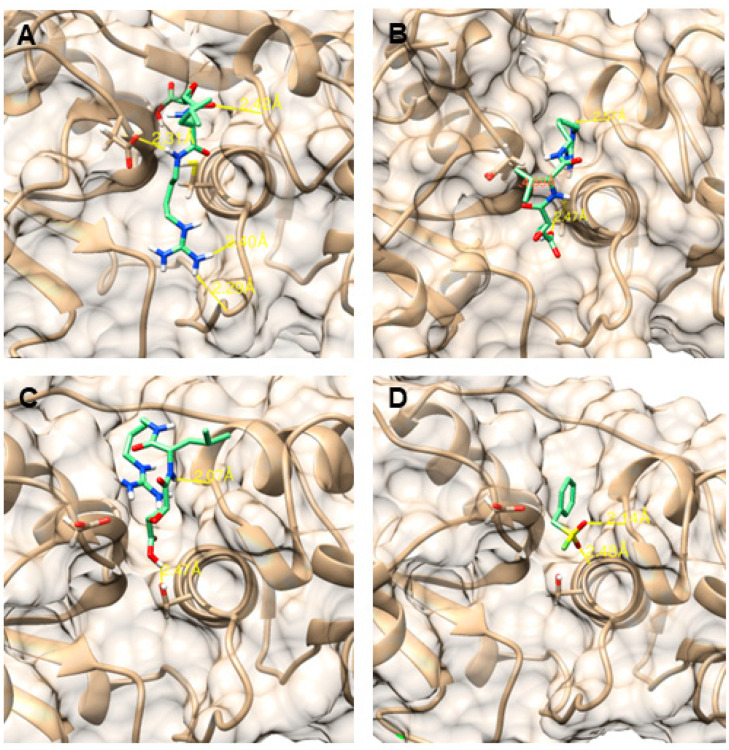
Static protein ligand docking between Sl-Cathl, Sl-CathL-CS, Sl-CathL-mutSC, and inhibitors. (**A**) Interaction between Sl-CathL and E-64. E-64 fits Sl-CathL active site as described for other cysteine cathepsins. (**B**) Sl-CathL-mutSC and E-64 docking. E-64 forms three bonds (depicted by yellow line) with active site of Sl-CathL-CS. Orange line indicates expected interaction between the two molecules, but they are too far for it to happen. (**C**,**D**) Static docking between Sl-CathL-CS and E-64 and between Sl-CathL-CS and PMSF. Neither inhibitor can form interactions with Sl-CathL-CS, as described for other proteases. Proteins are shown in gray, e inhibitors are colored according to their atoms, with C in green, N in blue, O in red and S in yellow. The distances between two atoms are depicted in Å and represented by yellow lines.

Static protein ligand docking was performed to better understand the differences in the activity of Sl-CathL-CS and Sl-CathL-mutSC. Due to the fact that Sl-CathL is a cysteine protease that shares a identity with both proteins, it was chosen as a control model (Figure 5A). E-64 fits better in Sl-CathL-mutSC (Figure 5B) than Sl-CathL-CS (Figure 5C) once it creates three interaction points with the first protein and two with the latter. Besides, E-64 does not assume the conformation described for other cysteine cathepsins when modelled in the putative Sl-CathL-CS active site. Since Sl-CathL-CS presents a serine on its putative catalytic site, we simulated a model of its interaction with PMSF (Figure 5D). However, PMSF did not assume the conformation described when it inhibited serine proteases.

## 3. Discussion

The initial analysis of the transcriptome of the larval midgut revealed around 1220 sequences related to Sl-CathL, which were further filtered by considering the identity percentage, and the final sequences were split into three groups according to their similarity with Sl-CathL. Group 1 shared between 75 and 99% similarity, group 2 shared between 50 and 74%, and group 3 shared between 30 and 49% (Figure 1). Groups 2 and 3 contained putative proteins that presented amino acid residues other than Cys^138^ in the expected catalytic triad, such as serine, threonine, arginine, and phenylalanine (Figure 1). The putative proteins that present arginine and phenylalanine are unlikely to show any protease activity, and this assumption is reinforced by the lack of an inhibitory propeptide region and H^272^, a component of the catalytic triad. We report the identification, recombinant expression, and characterization of Sl-CathL-CS, which despite sharing identity (47%) with Sl-CathL [2], presented a serine instead of the expected cysteine in the catalytic site. 

A gene expression analysis through RT-qPCR of the different larval body tissues displayed that Sl-CathL-CS was mostly expressed in the midgut, with the highest expression levels in 20- and 30-day-old larvae (Figure 2). These results exclude the possibility that Sl-CathL-CS is an isoform of a lysosomal cysteine cathepsin and, more importantly, indicate that Sl-CathL-CS is necessary only when the insect is feeding on the plant, since the adults live outside the sugarcane on the ground of the plantations. Additionally, the high expression level in the midgut and the fact that the Sl-CathL-CS expression pattern prove similar to that of Sl-CathL [2] and are indicative of the auxiliary role of the protein in the digestive process.

Relationships between plants and their insect predators are good examples of a fine-tuned co-evolutionary process. Insects produce a rich arsenal of enzymes, including digestive proteases, while host plants produce micromolecules and protein inhibitors [21,22,23]. Phytocystatins (PhysCys) are plant protease inhibitors involved not only in several developmental roles but also in plant protection against abiotic stress (e.g., drought, mechanical damage) and biotic stress (e.g., predation) [24,25]. Many PhysCys may prevent herbivory by inhibiting the insects’ main digestive enzymes, including cysteine proteases [25,26,27,28]. 

Plant protease inhibitors (PIs) can positively select and alter the expression profiles of digestive proteases in the insect midgut. In several Lepidoptera, a diet containing serine protease inhibitors led to the expression of more types of digestive proteases, which were both sensitive and insensitive to the inhibitors [29,30]. The addition of engineered tomato cystatin SlCY8 and its variants, cystatin D, and oryzacystatin I and II, to the beetle *Leptinotarsa decemlineata* diet altered the profile expression of digestive proteases, which increased the expression of cysteine and other proteases (aspartic and serine proteases) that are insensitive to the inhibitors [31,32,33]. Accordingly, an analysis at the molecular level of the *L. decemlineata* digestive process revealed that insects that fed on inhibitors produced different enzymes that were structurally similar to those found in the control group, however, they differed by up to 50% in sequence identity [34]. Insect susceptibility to PI is influenced by the insect’s natural protease arsenal: species that naturally produce a broader array of proteases are less affected by a chronic diet containing PI, and increased protease gene expression seems to play a major role in this scenario [35]. The compensatory strategies insects utilize when fed with PI are as follows: an increased expression of the target proteases to compensate for the inhibition, the production of proteases that are insensitive to the inhibitors, the production of proteases that target or neutralize the PI, or the expression of isoforms through selective pressure. Insects more often use a combination of these [36]. Sl-CathL-CS can fit into the fourth case as a protease isoform that can interact with inhibitors and impede them to inhibit the true proteases.

To further investigate this theory, we designed and expressed the site-direct mutated protein Sl-CathL-mutSC, related to Sl-CathL-CS, in which the Ser^138^ was reverted to a cysteine residue. Surprisingly, even though the only difference between cysteine and serine amino acids is reliance on their nucleophile side chain (while cysteine presents a thiol group, serine presents a hydroxyl group), the two proteins presented different activities. The first difference was noted during protein expression. Sl-CathL-CS reached the highest expression level in 24 h (Figure 3A), as indicated by one diffuse band around 45 kDa in SDS-PAGE. This pattern is expected when *P. pastoris* secretes proteins to the medium. Sl-CathL-mutSC achieved its maximum protein yield within 48 h of induction (Figure 3B), and its production is shown with a darker band with around 35 kDa, in a pattern with different molecular weights. This pattern was noticed by our group before, and is an indication that the protease maturates during induction and it is degrading proteins from *P. pastoris*, because cysteine proteases are activated by the acidic pH, which is a consequence of long periods of protein induction. Therefore, this is the first result indicating that Sl-CathL-mutSC possesses proteolytic activity while Sl-CathL-CS does not. Sl-CathL-CS showed no activity on either a complex substrate (skim milk powder, Supplementary Figure S4) or the specific fluorometric substrates for serine or cysteine proteases. On the other hand, Sl-CathL-mutSC was capable of degrading skim milk powder (Supplementary Figure S4) and Z-Phe-Arg-AMC (Figure 3C), but not Z-Leu-Arg-AMC and Z-Arg-Arg-AMC. These results indicate that Sl-CathL-mutSC shows the expected hydrolytic activity of cysteine cathepsin L [37]. To confirm cysteine protease activity, we tested Sl-CathL-mutSC with E-64 and PMSF, specific cysteine and serine proteases inhibitors, respectively [38,39]. As expected, E-64 was able to inhibit Sl-CathL-mutSC (Figure 3D), while the addition of PMSF caused no inhibition.

The next step was to evaluate the interactions between Sl-CathL-CS and Sl-CathL-mutSC and CaneCPI-1, a sugarcane cystatin, for which the overexpression in sugarcane was proven to inhibit *S. levis* larval growth in planta [40]. The change of serine to cysteine alters the capability of the protein to interact with CaneCPI-1. In Figure 4, we compared the binding profile of Sl-CathL-CS and Sl-CathL-mutSC with CaneCPI-1. In this assay, we carried out two washing and two elution steps, the latter containing 250 mM of imidazole. Lane 3 for the assay with Sl-CathL-CS (Figure 4A) and lane 11 for the assay with Sl-CathL-mutSC (Figure 4B), both of which corresponded to the second washing step, should be highlighted. A protein band corresponding to CaneCPI-1 is evident in the assay with Sl-CathL-mutSC (lane 11, Figure 4B), while it is not detected in in an assay conducted using Sl-CathL-CS (lane 3, Figure 4A). This finding indicates that CaneCPI-1, when incubated with Sl-CathL-mutSC, is eluted without the addition of imidazole, as detected in the washing step. On the other hand, the cystatin interacts strongly with Sl-CathL-CS, once CaneCPI-1 is detected when Sl-CathL-CS is eluted with imidazole. The Western blotting performed with the anti-CaneCPI-1 antibody and the anti-His antibody confirmed the absence of CaneCPI-1 in the washing steps of the assay with Sl-CathL-CS, and the detection of CaneCPI-1, exclusively in the elution steps indicating that it strongly interacts with Sl-CathL-CS, and is eluted only when the latter is no longer adsorbed in the column. As shown at the bottom of Figure 5, the Western blotting conducted with the anti-His antibody provides evidence that the His-tag was efficiently removed from CaneCPI-1 and that the detection of this cystatin in the elution steps was due to its binding to Sl-CathL-CS and Sl-CathL-mutSC.

Static protein ligand docking illuminates the differences in the putative catalytic triad of Sl-CathL-CS and Sl-CathL-mutSC. Sl-CathL was used as a model (Figure 5A) because it is a known cysteine cathepsin that is inhibited by E-64, as described for other proteases in this class. E-64 fits the oxyanion hole of Sl-CathL-mutSC (Figure 5B), but with fewer interaction points when compared to the model of E-64 and Sl-CathL. Accordingly, E-64 presents higher K_i_ to Sl-CathL-mutSC than to Sl-CathL. E-64 (Figure 5C) and PMSF (Figure 5D) did not fit Sl-CathL-CS with the conformation described for cysteine cathepsins and serine proteases. These results corroborate that Sl-CathL-CS is a pseudoenzyme.

A mutation from cysteine to serine residues has been reported in different proteins from several organisms, from bacteria to human, affecting the protein role. A single mutation from a nucleotide leading to the translation of serine instead of cysteine in mouse tyrosinase results in the albino phenotype [41]. In *Salmonella typhimurium*, the change of cysteine to serine in the sulfate binding protein causes an affinity loss of greater than 3000 times. Although the modification is considered to be conservative, the difference of volume and, especially the angle conformation between thiol and hydroxyl groups, can explain the substantial affinity loss between the sulfate binding protein and sulfate [42]. Likewise, the substitution of serine for cysteine residues in tryptophan repressors in different positions leads to its decreased affinity to tryptophan and/or DNA, influencing the operation of tryptophan operon in *E. coli* [43]. The serine-to-cysteine mutation in β-lactamase of *Bacillus licheniformis* destabilizes the protein core, causing folding alteration [44].

Considering the results presented, along with the context of insect plant interactions, Sl-CathL-CS is likely to play an accessory role in *S. levis* larvae digestion, interacting with cystatins and allowing Sl-CathL to operate as the major digestive protease.

## 4. Materials and Methods

### 4.1. RNA Extraction

The different parts (head, fat body, midgut, hindgut, hemolymph, and carcass) of 30-day-old *S. levis* larvae (*n* = 4) and entire individuals (*n* = 4) in several development stages (eggs; 10-, 20- and 30-day-old larvae; prepupa; pupa; and adults) were used to extract total RNA with TRIzol Reagent (Invitrogen, Carlsbad, MA, USA), according to the manufacturer’s instructions.

### 4.2. Library Preparation and Illumina Sequencing

The midgut RNA from 30-day-old larvae (n = 4) was treated with RNAse-free DNAse I (Invitrogen, Waltham, MA, USA) in reactions with 3 μg of RNA to a final volume of 20 μL. Poly (A) mRNA was isolated with oligo(dT) beads and transcriptome construction was conducted with TruSeq Small RNA Library Preparation Kits (Illumina, San Diego, CA, USA), according to the manufacturer’s instructions. The mRNA was fragmented in a Fragmentation Buffer 1 × (Illumina, San Diego, CA, USA) and used as a template in a reaction with SuperScript II (Invitrogen, Waltham, MA, USA) with random hexamer primers to obtain the first-strand cDNA, followed by a reaction with DNA polymerase I and RNAse H (Illumina, San Diego, CA, USA) to synthesize the double-strand cDNA. After pair-ending (2 × 100 bp), adenylation, and ligation, suitable fragments (200 ± 30 bp) were selected and sequenced using HiSeq1000 (Illumina, San Diego, CA, USA). The raw data from the sequencing runs were submitted to the Sequence Read Archive (SRA) repository of the National Center for Biotechnology Information under BioProject number PRJNA694547, BioSample: SAMN17526444 and SRR13518685.

### 4.3. Quality Control, Preprocessing and de Novo Assembly

The raw read quality control was obtained using the FASTQC v.0.11.9 program [45]. Trimmomatic version 0.36 [46] was used for trimming the first 15 bp (HEADCROP:15 parameter), and a de novo assembly was performed with Trinity version 2.9 [47]. We evaluated the transcriptome completeness using Benchmarking Universal Single-Copy Orthologs (BUSCO) v4.0.4 [48]. This analysis provided a quantitative and comprehensive overview of the level of completeness for our assembly. The transcriptome was compared with a predefined set of Eukaryota single-copy orthologs from the OrthoDB v10 database. Contigs were categorized as “complete, single copy”, “complete, duplicated copy”, “fragmented”, or “missing”, depending on the length of the aligned sequence.

### 4.4. Identification of Putative Pseudo-Cysteine Proteases

To identify putative enzymes in *Sphenophorus levis* midgut transcriptome we used the OmicsBox (BioBam, Valencia, Spain) platform to run the local BlastP tool (cut off e-value 1e-03) against a local database with the Sl-CathL sequence as reference. Approximately 1220 sequences were retrieved, and we used a minimum coverage of 40% to filter the fragments. Then, sequences were individually analyzed and the putative protein that presented the Cys^138^ and His^272^, which are two residues from the catalytic triad, were chosen for further exploration. The final sequences were divided into three groups, according to their shared identity with Sl-CathL: group 1, between 99 and 75% identical to Sl-CathL; group 2 between 74 and 50% shared identity; and group 3, between 49 and 30%. In group 3, we identified one sequence that presented Ser^138^ instead of Cys^138^, and one was named Sl-CathL-CS. The identity matrix was calculated using Clustal Omega [49].

### 4.5. Relative Gene Expression of Sl-CathL-CS in Different Developmental Stages and Parts of the Larvae

Approximately 600 ng of pooled RNA was treated with DNAse I (Invitrogen, Waltham, CA, USA) and used to synthesize cDNA in a reaction using the ImProm-II Reverse Transcription System (Promega, Madison, WI, USA) with 0.5 μg of Oligo(dT) (Promega, Madison, WI, USA) in a 20 µL reaction. The primers for Sl-CathL-CS and glyceraldehyde 3-phosphate dehydrogenase (GAPDH), that were used as the internal calibrator, were designed with the aid of the Primer3 program, version 4.0 [24], and are listed in Table 2. A real time reverse transcription quantitative PCR (RT-qPCR) was conducted using 5 μL of Platinum SYBR Green qPCR Supermix (Invitrogen, Waltham, CA, USA), 10 × diluted cDNA and non-template control, and 0.4 μM of each primer in a 10 μL reaction in 3 technical replications. The thermal cycling conditions were 50 °C for 2 min and 95 °C for 30 s, followed by 40 cycles of 54 °C for 30 s, 72 °C for 40 s, and the melting curves were obtained at 95 °C for 15 s, 55 °C for 15 s and 95 °C for 15 s. The data were analyzed according to the 2^−ΔΔCT^ method [50]. For the relative expression of the developmental stages, the Sl-CathL-CS gene expression in pupa was used as a reference, because it exhibited the lowest expression. To determine the relative expression of Sl-CathL-CS in parts of the larvae, midgut levels were used as a reference.

### 4.6. Heterologous Expression of Sl-CathL-CS and Sl-CathLCS-mutSC

The open reading frame (ORF) for the putative protease Sl-CathL-CS (GenBank: JZ136744.1) was used for designing primers and cloning Sl-CathL-CS into the expression vector pPICZαA. Forward and reverse primers (Table 2) were designed with restriction sites for *Eco*RI and *Not*I (indicated in bold), respectively. The signal peptide was identified using the SignalP 4.1 server [26] and then excluded. The PCR was conducted using around 50 ng of the template DNA from the one cDNA library previously made in our laboratory, 200 µM of dNTPs, 1.5 mM MgCl_2_, 10 pM of each primer, 1 × PCR buffer, and 1 U of Platinum Taq DNA Polymerase High Fidelity (Invitrogen, Waltham, CA, USA) in a 25 μL reaction. The reaction began with the denaturation for 1 min at 94 °C, followed by 35 cycles of 1 min at 94 °C, 1 min at 50 °C, and 1 min at 72 °C with final extension for 10 min at 72 °C. The amplicon was analyzed in 1% agarose gel and the product was digested with *Eco*RI and *Not*I and cloned into a pPICZαA vector (Invitrogen, Waltham, CA, USA) cleaved with the same enzymes, resulting in the plasmid pPICZαA-Sl-CathL-CS. 

The reversion of Ser^138^ to Cys^138^ was performed using the Quick-Change II Site-directed Mutagenesis kit (Agilent, Santa Clara, CA, USA) to generate plasmid pPICZαA-Sl-CathL-mutSC. Primers Sl-CathL-mutSCforw and Sl-CathL-mutSCrev (Table 2), containing a codon for cysteine (underlined in the sequence of primers), were designed, and pPICZαA-Sl-CathL-CS was used as the template. The mutation was confirmed by dideoxy termination sequencing. The linearization of plasmids pPICZαA-Sl-CathL-CS and pPICZαA-Sl-CathL-mutSC with the restriction enzyme *Pme*I, the transformation in *P. pastoris* KM71H strain, and the colony screening were all performed as previously described [2].

### 4.7. Enzymatic Characterization of Sl-CathL-CS and Sl-CathL-mutSC

#### 4.7.1. Sl-CathL-CS and Sl-CathL-mutSC Protease Activity

First, Sl-CathL-CS and Sl-CathL-mutSC protease activity was explored using a complex substrate. Around 1.5 µg of each protein was incubated with 0.5% (*w*/*v*) skim milk powder and the sodium acetate buffer (10 mM, pH: 4.5), to a final volume of 1 mL, at 37 °C for 24 h. Aliquots were obtained every hour and protein degradation was analyzed in 15% SDS-PAGE [51]. 

The next stage included assessing the activity of the two proteins on specific serine and cysteine protease substrates. This assay was performed as described before [2] with a few modifications. The Sl-CathL-mutSC activity was measured in a Hitachi F-2500 spectrofluorometer (Hitachi, Tokyo, Japan) by measuring the substrate groups AMC and EDDnp release, which fluorescence can be monitored at λ_ex_ = 380 nm and λ_em_ = 460 nm. Approximately 4.3 nM of the proteins was incubated with 4 mM of DTT in a 500 μL reaction with sodium acetate 10 mM buffer pH: 5.0 for 5 min at 37 °C. The proteolysis was measured using increasing concentrations (40 µM to 1.2 mM) of cysteine cathepsins fluorogenic substrates Z-Phe-Arg-AMC (Calbiochem, San Diego, CA, USA), Z-Leu-Arg-AMC (Calbiochem, San Diego, CA, USA ), and Z-Arg-Arg-AMC (Calbiochem, San Diego, CA, USA) for both proteins, and the Sl-CathL-CS was tested on serine protease substrates Abz-Lys-Leu-Phe-Ser-Ser-Lys-Gln-EDDnp (Sigma-Aldrich, St. Louis, MO, USA), Abz-Phe-Ser-Lys-Gln-EDDnp (AminoTech P&D Ltda, Diadema, Brazil), Abz-KNRSSKQ-EDDnp (AminoTech P&D Ltda, Diadema, Brazil), and Abz-Gly-Ile-Val-Arg-Ala-Lys-Gln-EDDnp (AminoTech P&D Ltda, Diadema, Brazil).

Both E-64, a classic cysteine protease inhibitor, and PMSF, a serine protease inhibitor, were also tested against Sl-CathL-mutSC. Increasing concentrations of E-64 (10–100 μM) were tested and the inhibition constant (K_i_) was calculated according to Morrison [52] using GraFit software, version 5.0 [53].

#### 4.7.2. Competition Assay

The interaction between Sl-CathL-CS and sugarcane cystatin CaneCPI-1 was evaluated in a competition assay. The proteolytic activity of Sl-CathL (10 nM) on substrate Z-Phe-Arg-AMC (20 mM) was measured as described in the previous section through a kinetic reaction measured in a Hitachi F-2500 spectrofluorometer. Then, CaneCPI-1 (5 nM) was added to the reaction and the remaining proteolytic activity of Sl-CathL was calculated. Increasing concentrations of Sl-CathL-CS (0.01–0.3 μM) were mixed to the reaction and the re-establishment of the protease was measured. The assay was performed in triplicate and the protease activity is presented as relative activity (%). Statistical analyses were performed with the aid of GraphPad Prism software, version 6.00 (La Jolla, CA, USA).

### 4.8. Pull-Down 

The CaneCPI-1 was produced and purified as previously described [19]. One unit of thrombin (Abcam, Cambridge, UK) was added to 250 μg of CaneCPI-1 in a 500 µL reaction in a PBS buffer (pH 8.0) at room temperature, for 2 h, to remove the 6x His-tag. Approximately 2.5 µg of each of the purified proteins Sl-CathL-CS and Sl-CathL-mutSC was mixed separately with 250 μg of CaneCPI-1 (without the His-tag), 50 µL of Ni-NTA agarose (Invitrogen, Waltham, MA, USA), and sodium acetate buffer solution (10 mM, pH: 5.5) in a final volume of 1 mL. The reaction was incubated on ice for 2 h with gentle shaking. The supernatant was removed after centrifugation for 1 min at 13,000× *g*, followed by the 2 washing steps with 200 µL of the lysis buffer (100 mM NaCl, 50 mM NaH_2_PO_4_, 10 mM Tris; pH: 8.0). Finally, the elution was performed twice with 200 μL of lysis buffer with 250 mM of imidazole. Samples were analyzed in SDS-PAGE 15% and Western blotting. 

The Western blotting was conducted using 2 primary antibodies: monoclonal antibody anti His-tag (GE Life Sciences, Marlborough, MA, USA) and polyclonal antibody anti-CaneCPI-1 [19]. The proteins were transferred to the Amersham Hybond 0.45 µm PVDF membrane (GE Life Sciences, Marlborough, MA, USA), in the Mini Trans-Blot Electrophoretic Transfer Cell (Bio-Rad, Hercules, CA, USA) for 2 h under 150 mA. The membrane was blocked with 5% (*w*/*v*) skim milk powder in TBS buffer (150 mM NaCl, 50 mM Tris–HCl, pH 8.0) and washed with a TBS buffer. Then, the membranes were incubated with an anti-His antibody (1:5000 dilution; GE Life Sciences, Marlborough, MA, USA) or anti-CPI-1 antibody (1:5000 dilution) and washed with a TBS buffer. The membranes were incubated with an anti-Mouse IgG Antibody (1:5000 dilution; KPL, New Delhi, India), washed, and detected with a Clarity Max ECL Substrate (Bio-Rad, Hercules, CA, USA).

### 4.9. Static Protein-Ligand Docking

The structures of Sl-CathL, Sl-CathL-CS, and Sl-CathL-mutSC were simulated by MODELLER [54] using the default settings. Both E-64 and PMSF were extracted from Pubchem (codes 123985 and 4784, respectively) using USCF Chimera 1.15 [55]. All of the structures had their energy minimized and were optimized for analysis. They were submitted to static docking in the UCSF Chimera 1.15 program with the aid of AutoDock Vina [56]. The modeling was performed with an exhaustiveness = 8 and a difference of energy for the model determination = 2 kcal/mol.

## Figures and Tables

**Table 1:** *S. levis* larvae midgut transcriptome preprocessing and assembly.

Number of raw reads	70,720,844
Number of readings after filtering	146,145
Average length of contigs (bp)	1131.23
N50 length (bp)	2382
GC Percent	37.20

**Table 2:** Primers used for plasmid construction and RT-qPCR analysis. The restriction sites are in bold and the modified codon is underlined.

Primer	Sequence (5′–3′)	Purpose	Amplicon (bp)
Sl-CathL-CS_fw:	CC**GAATTC**AGTTCGGAGCTGAACATGG	ORF cloning	984
Sl-CathL-CS_rv	ATTCTTAT**GCGGCCGC**ATCGATTTCGACGTAGGCAGC		
Sl-CathL-mutSCforw	ACCAAGGAGAATGGGATACATGTTGGGCTTTCTCCACTATTGC	Site-directed mutagenesis	
Sl-CathL-mutSC_rev	GCAATAGTGGAGAAAGCCCAACATGTATCCCATTCTCCTTGGT		
Sl_GADPH_qF	CAACTGGCGTTTTTACCACA	Real Time PCR	104
Sl_GADPH_qR	AACATACATTGGGGCGTCA		
Sl_CathL-CS_qF:	ATACGACTGGAGGGAGCAGA	Real Time PCR	108
Sl_CathL-CS_qR	ATGGCGTAGGCACTTTCAAC		

## Data Availability

Raw data from the sequencing runs were submitted to the Sequence Read Archive (SRA) repository of the National Center for Biotechnology Information under Bioproject number PRJNA694547, BioSample: SAMN17526444 and SRR13518685.

## Supplementary Materials

The following are available online at https://www.mdpi.com/article/10.3390/ijms222111476/s1.
