# Supplementary material for: Identification and Functional Analysis of a Pseudo-Cysteine Protease from the Midgut Transcriptome of Sphenophorus levis
Source: Int J Mol Sci. 2021 Oct 25;22(21):11476. doi: 10.3390/ijms222111476 (PMC8583781; doi:10.3390/ijms222111476)
Supplement: Supplementary file 1 [file ijms-22-11476-s001.zip › ijms-1385503-supplementary.pdf]

Supplementary material 1

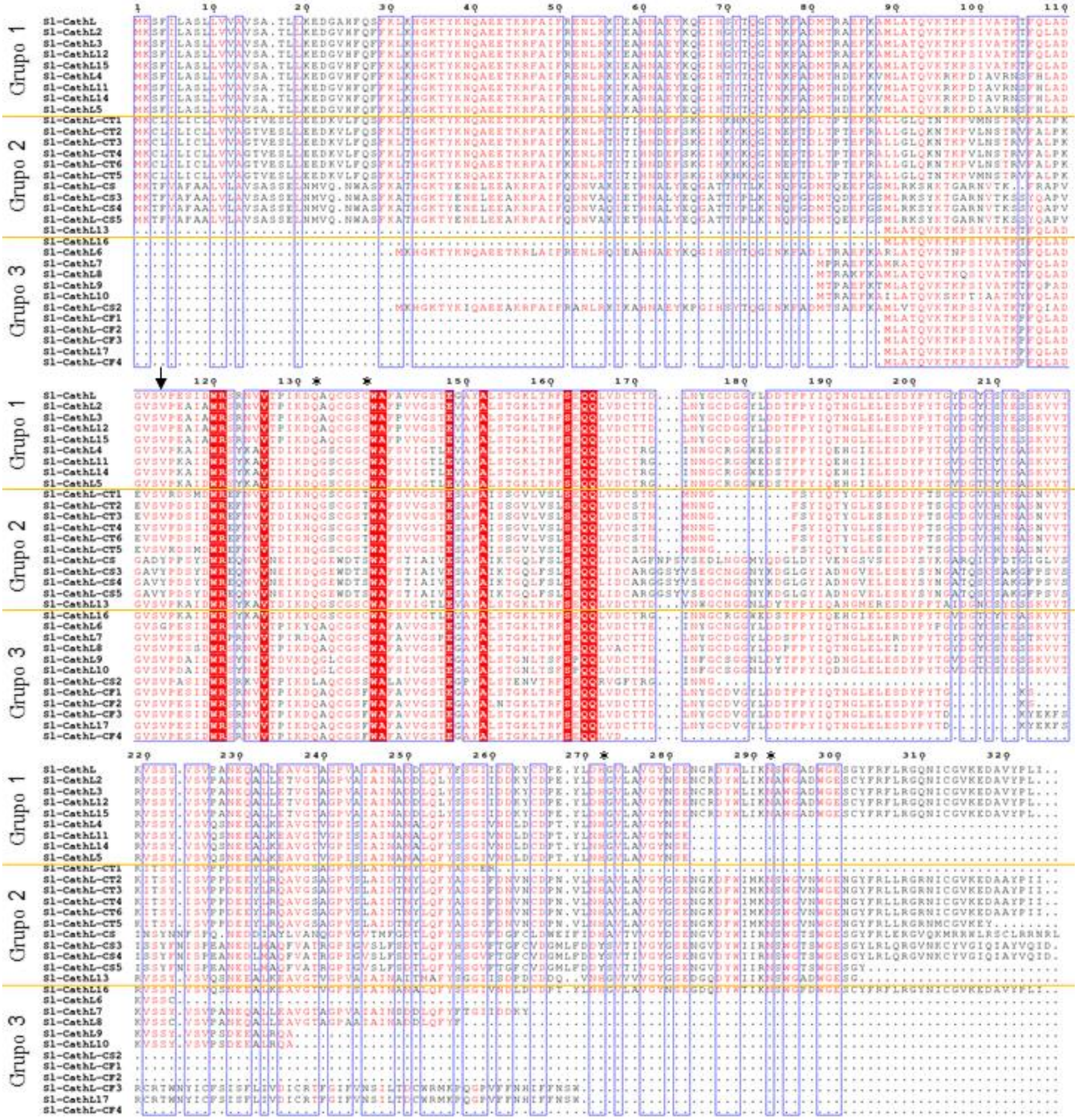

Supplementary Figure S1. Alignment of Sl-CathL and other variants in *S. levis* 30-day-old larvae midgut transcriptome. Sequences were divided into 3 groups according to the identity with Sl-CathL. Asterisks indicate residues forming the catalytic triad. The arrow indicates the putative propeptide cleavage site. Residues in red share high consensus, while the ones in blue are the most variable. Identical residues are highlighted in red and the blue box indicates similar parts of the protein sequence.

Supplementary material 2

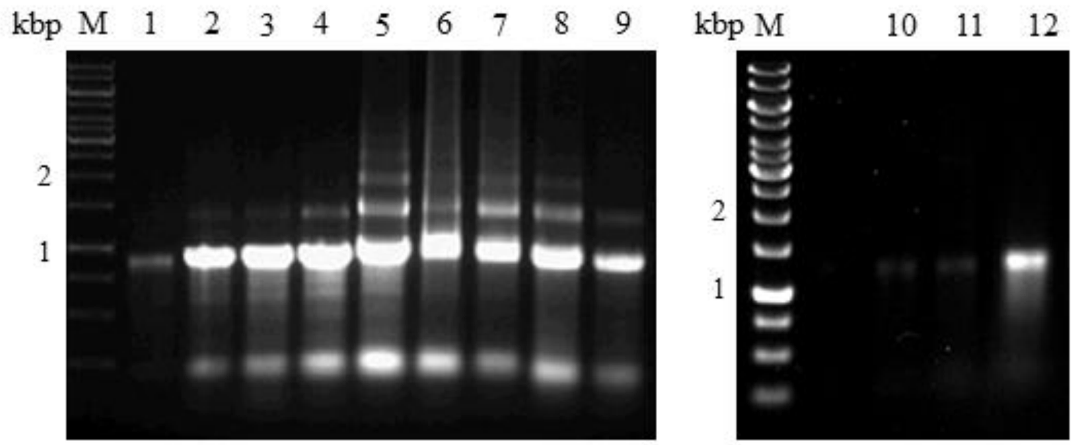

Supplementary Figure S2. RNA used for gene expression analysis. Insect RNAs contain a hidden break in 28S rRNA and run in a similar pattern as 18S rRNA. M: GeneRuler 1 kb (Thermo Scientific). 1: eggs, 2: 10-day-old larvae, 3: 20-day-old larvae, 4: 30-day-old larvae, 5: prepupa, 6: pupa, 7: midgut, 8: hindgut, 9: hemolymph, 10: integument, 11: fat body, 12: head. Samples 10 – 12 were already treated with DNAse, and samples 1 – 9 were not.

Supplementary material 3.

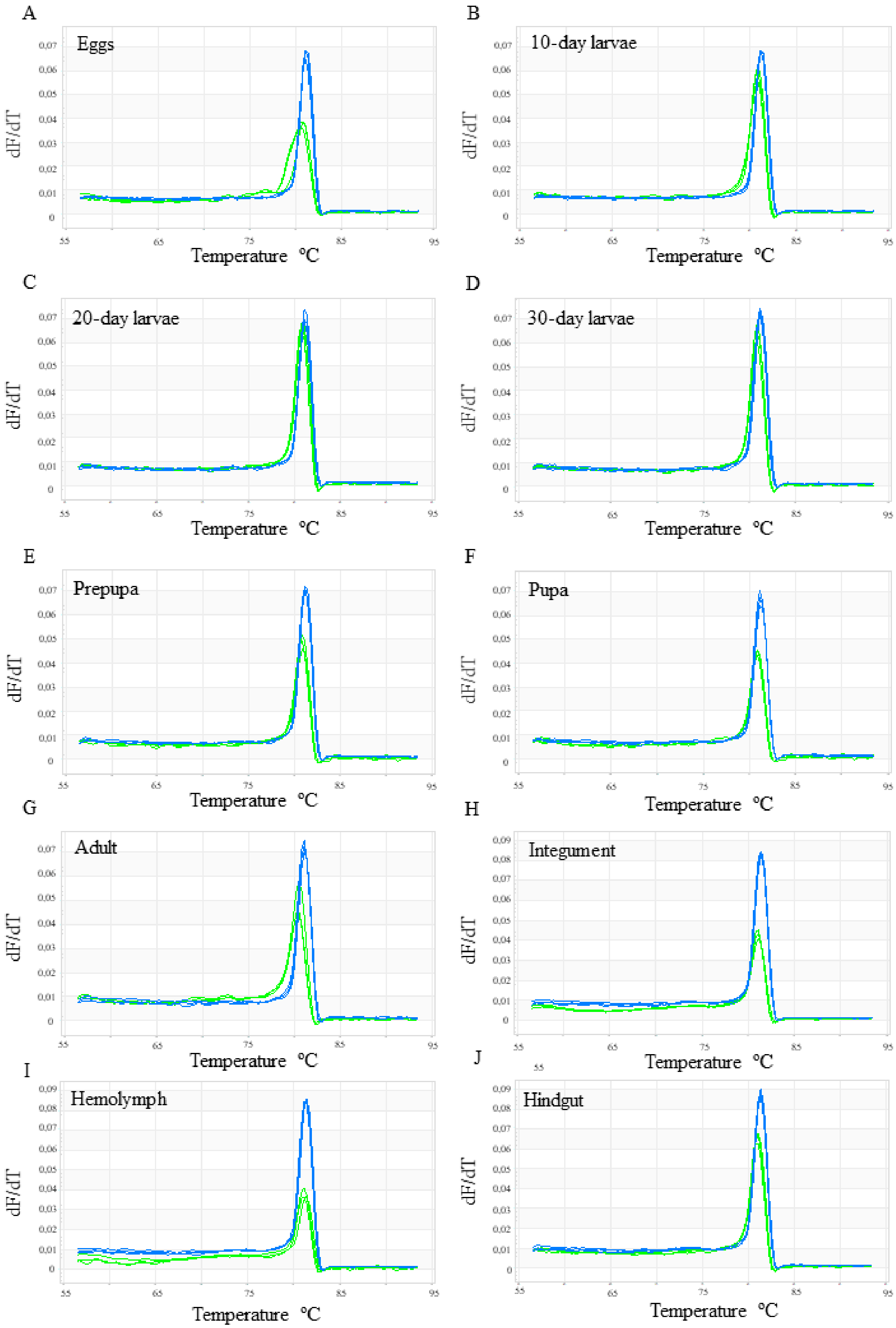

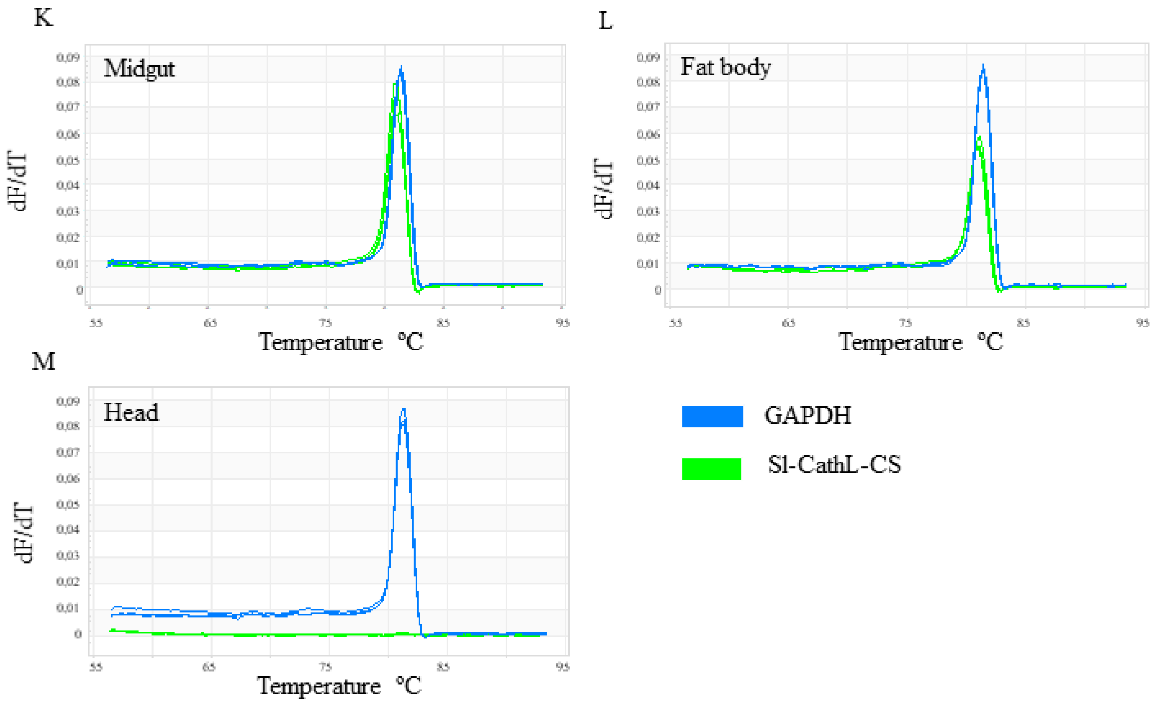

Supplementary Figure S3. Derivative melting-curve analysis of GAPDH and Sl-CathL-CS genes in *S. levis* developmental stages and tissues. Blue line indicates GAPDH and the green line shows Sl-CathL-CS. Similar peaks indicates no primer-dimer formation and absence of contamination. RT-qPCR analysis was carried out in different developmental stages and 30-day-old larvae tissues, being A: eggs, B: 10-day old larvae, C: 20-day old larvae, D: 30-day old larvae, E: prepupa, F: pupa, G: adult, H: integument, I: hemolymph, J: hindgut, K: midgut, L: fat body, M: head.

Supplementay material 4

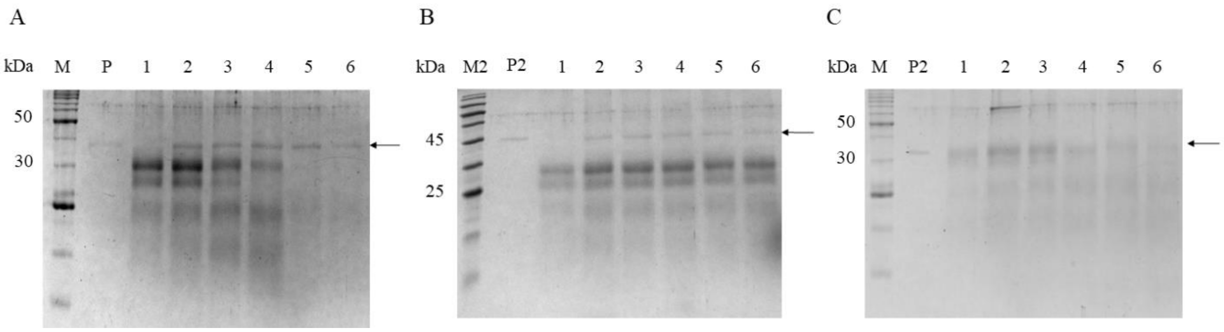

Supplementary Figure S4. Enzyme activity of Sl-CathL-CS and Sl-CathL-mutSC. (a) Sl-CathL (b) Sl-CathL-CS and (c) Sl-CathL-mutsSC with powder milk. P, P1 and P2 indicate the respective proteins, lane 1 is the powder milk solution and lanes 2, 3, 4, 5 and 6 are the samples collected after 0, 1, 2, 4 and 8 hours, respectively. The black arrows indicate the proteins. M: Benchmark Protein (Thermo Fisher); M2: Prestained Protein Marker MW (Jena Biosciences).
